# Towards ‘Formalising’ WhatsApp Teledermatology Practice in KZ-N District Hospitals: Key Informant Interviews

**DOI:** 10.3390/ijerph21101388

**Published:** 2024-10-21

**Authors:** Christopher Morris, Richard E. Scott, Maurice Mars

**Affiliations:** 1 Department of TeleHealth, School of Nursing & Public Health, College of Health Sciences, University of KwaZulu-Natal, Durban 4041, South Africa; ntc.ehealthconsulting@gmail.com (R.E.S.); mars@ukzn.ac.za (M.M.); 2 Department of Community Health Sciences, Cumming School of Medicine, University of Calgary, Calgary, AB T2N 4N1, Canada

**Keywords:** instant messaging, WhatsApp, smartphones, teledermatology, South Africa, key informant interviews

## Abstract

Introduction: District hospitals in KwaZulu-Natal Province, South Africa, do not have onsite specialist dermatology services. Doctors at these hospitals use WhatsApp instant messaging to informally seek advice from dermatologists and colleagues before possible referral. They have expressed the need to formalise WhatsApp teledermatology. Aim: To determine the views and perspectives of clinicians on the feasibility and practicality of formalising the current WhatsApp-based teledermatology activities within the KwaZulu-Natal Department of Health Dermatology Service. Methods: Key informant interviews with 12 purposively selected doctors at district hospitals and all 14 dermatologists in the KwaZulu-Natal dermatology service. Their views and perspectives on formalising the current informal use of WhatsApp for teledermatology were recorded, transcribed, and thematically analysed. Results: Five primary themes (communication, usability, utility, process, and poor understanding of legal, regulatory, and ethical issues) and 22 sub-themes were identified. Clinicians wanted WhatsApp teledermatology to continue, be formalised, and be incorporated within the KwaZulu-Natal Department of Health, facilitated by the provision of practical guidelines addressing legal, regulatory, and ethical issues. Conclusions: These findings will be used to develop a policy brief, providing recommendations and proposed guidelines for formalising the teledermatology service. The findings and methods will be relevant to similar circumstances in other countries.

## 1. Introduction

Telemedicine is defined as the use of Information and Communication Technologies (ICT) to provide healthcare over distance [1]. This includes all forms of electronic communication, ranging from a simple telephone call between healthcare providers to highly complex applications such as remote-controlled surgery [2]. The COVID-19 pandemic caused a significant shift towards telemedicine use by clinicians to enable access to and continuity of care for patients whilst maintaining social distancing requirements. It accelerated the adoption of digital technologies used in telemedicine, including smartphones and instant messaging apps (such as WhatsApp) and videoconferencing apps (such as Skype and Zoom) for clinical communication and remote consultation [3]. Such increased awareness and applications encourage routine use of telemedicine. 

Africa needs telemedicine to help overcome its shortage of doctors and specialists and poor infrastructure (especially in rural areas) and to strengthen health systems and provide universal health coverage to its primarily rural populations [1]. South Africa was an early adopter of telemedicine, both synchronous and asynchronous. The National Department of Health (DoH) launched the National Telemedicine System in the late 1990s [4], but this top-down approach mostly failed within two years for a variety of reasons, and telemedicine in the country stagnated [5]. Telemedicine activities were, however, introduced at several medical schools, including store and forward teledermatology [6,7], pathology services [8], video-conferenced teledermatology [9], and tele-education [10]. The challenges faced in South Africa, common to most African countries, have led to low uptake of telemedicine on the continent [11,12]. 

In the absence of formally established telemedicine programmes, doctors in South Africa and other African countries have found a way of delivering health services themselves using instant messaging (IM)-based apps and smartphones [13,14,15,16,17], often paying for the connectivity and communications costs themselves [18]. A recent survey of district hospitals in KwaZulu-Natal (KZ-N), South Africa, reported widespread use of WhatsApp by clinicians for teledermatology and other specialities [19]. The district hospitals are a component of the National Health system in South Africa that serves approximately 84% of the population [20]. These hospitals do not have resident dermatologists and refer dermatology patients who require specialist opinion to regional or tertiary facilities in the province. The informal use of IM in these hospitals has improved access to specialist healthcare in rural areas and contributed to substantial time and cost savings for both the physician and the patient [19]. 

The Health Professions Council of South Africa (HPCSA) is a statutory body that guides good clinical practice in South Africa. In 2014, they published The General Ethical Guidelines for Good Practice in Telemedicine [21]. These guidelines were developed before the widespread adoption of smartphones and IM apps by clinicians were not well publicised, and some terms used were not clearly defined. Although revised in 2021 to allow remote management of patients in response to COVID-19 [22] they do not adequately address the use of instant messaging. Recent South African studies have shown that most clinicians were either unaware of HPCSA guidelines or disregarded them despite the potential risks [16,23], a trend noted elsewhere in the world [24]. Similarly, while the HPCSA has produced guidelines for the use of social media by doctors [25], these do not clearly take into account the use of IM (i.e., WhatsApp) for inter-clinician consultation, and a 2019 literature review found no guidelines for WhatsApp telemedicine [26]. More recently, in December 2022, the National Health Service in England published guidelines for using mobile messaging “where there is no practical alternative and the benefits outweigh the risks” [27].

The rationale for this study was threefold. First, doctors in the district hospitals in KZ-N had previously indicated their preference for WhatsApp teledermatology to be formalised and proceed as an approved service [19]. Second, before altering a functioning current practice, it is essential to understand the circumstances prevailing in any particular setting and determine how best to formalise consultations using IM apps. Third, the study was undertaken at the request of the KZ-N Department of Health eHealth Steering Committee. The study does not explore the direct medical aspects of service delivery but focusses on policy-related issues it is necessary to consider in order to formalise the current process.

The aims of this study were as follows: (i) to gain a more in-depth understanding of the informal use of WhatsApp by doctors and dermatologists within the public healthcare service provided by the KZ-N DoH, and (ii) to obtain the views and perspectives of doctors and dermatologists on formalising a WhatsApp-based teledermatology service. Such insight would not only help inform the KZ-N DoH and local healthcare practitioners about suitable operational, organisational, and staffing requirements to meet the need but also help inform similar practices in many other countries experiencing informal use of IM-mediated inter-professional consultation.

## 2. Methods

Key informant interviews (KII) were conducted in KZ-N between November 2021 and August 2022 to obtain the views and perspectives of doctors and dermatologists who informally use WhatsApp for teledermatology. In line with Hennink and Kaiser [28], a sample size of twelve doctors who had previously participated in a survey on the use of IM for teledermatology [19] was purposively selected. In addition, all 14 dermatologists in the KZ-N DoH service at the time of the study were interviewed. The development of the KII Guides, one for referring doctors and one for dermatologists, was based on the outputs of a previous survey of IM for teledermatology [19]. Prior to the interviews, the KII Guides were pre-tested in a trial interview with a doctor experienced in telemedicine and medical informatics. Questions were modified where necessary to avoid ambiguity. 

Similar KII guides were developed for both doctors and dermatologists. The KII Guides were intended to reveal the past and current practices of WhatsApp use by both doctors and dermatologists, covering four domains:

- WhatsApp use in the past—initial experiences
- Views of the current situation—the extent of WhatsApp use, related benefits, difficulties, concerns, and issues that need to be addressed
- Awareness of the HPCSA guidelines for telemedicine, including record-keeping and consent practices, and other legal, regulatory, and ethical (LRE) concerns in the use of WhatsApp
- Views of the future—suggestions for using on-call consultants, formalising and scaling up a WhatsApp-based teledermatology service and its integration into the dermatology service provided by the KZ-N DoH.

The KII were conducted by telephone (to abide by COVID-19 restrictions), recorded, and transcribed verbatim. A general thematic analysis was undertaken by the authors to identify and report patterns (themes) from across the data set following the six-phase guide of Braun and Clarke (data familiarisation, initial coding, theme development, theme review, define and name themes, report) [29]. Data were entered into a Microsoft Excel spreadsheet to help the authors code and identify themes deductively. After several iterations, the authors reached an agreement on the themes. The original analysis was driven by the research question and identified themes at the semantic level that were representative of issues confronting clinicians in their daily practice [30]. In addition, a second inductive analysis was undertaken to identify the underlying issues, concerns, and ideas that informed the semantic content of the data.

Iterative reading of the full set of individual and collective responses led to a better understanding of each respondent’s perspective. When feedback was vague or intention uncertain, responses were triangulated with those to other questions to clarify.

This study was undertaken at the request of the KZ-N DoH eHealth Steering Committee. Ethics approval was obtained from the University of KwaZulu-Natal (HSSREC/00001564/2020) and the KZ-N DoH ethics committees (NHRD Ref: KZ_2018_011). All respondents consented to participate. 

## 3. Results

Five primary themes and 22 sub-themes were identified after the second inductive thematic analysis (Table 1).

Sample quotations from the KII transcripts are provided in Appendix A—Detailed Results (Table A1, Table A2, Table A3, Table A4 and Table A5), where considered informative. These are indicative of the feedback from respondents (supportive and contradictory) and are from either a consulting doctor (Dr) or a dermatologist (Derma).

### 3.1. Theme 1—Communication

This theme concerned comments about the activity and people involved in communicating with one another to give or receive specific information. Subthemes included ‘initiation’ of WhatsApp use, ‘influence’ regarding initial introduction to use, ‘contacts’ made and by whom, the ‘frequency’ and ‘continuity’ of communication, and WhatsApp use for ‘other specialities’ (Appendix A, Table A1).

### 3.2. Theme 2—Usability

Comments regarding usability referred to the ease of use of WhatsApp to present a case history and clinical photographs. Sub-themes included: ‘Ease of use’, ‘Alternative IM apps’ used, and ‘Case history’ (Appendix A, Table A2).

### 3.3. Theme 3—Utility

Utility refers to comments noting the usefulness of WhatsApp (e.g., benefits clinical practice and patient care, cost-effective). Sub-themes included ‘Benefits’, ‘Connectivity’, ‘Status-quo’, ‘Learning, Education, Training’, and ‘Medication Access’ (Appendix A, Table A3).

### 3.4. Theme 4—Process 

Process refers to the actions or steps taken to achieve a particular end. For WhatsApp teledermatology consultation, this begins with contacting a dermatologist, ideally through a formalised and scaled procedure. Sub-themes included ‘On-call dermatologist’, ‘Formalising and scaling up’, and ‘What is wanted’ (Appendix A, Table A4).

### 3.5. Theme 5—Poor Understanding of LRE Issues

Legal and regulatory issues concern laws and rules that govern or impact the conduct of individuals within healthcare systems, whilst ethical issues involve rules or standards that (imposed through professional entities or social norms) govern the conduct of members of a profession. Sub-themes were ‘Privacy’, ‘Security’, and ‘Other LRE issues’. Notable sub-sub-themes were *‘Responsibility for patient care’*, *‘Consent’*, *‘Record-Keeping’*, *and ‘Guidelines—HPSCA’* (Appendix A, Table A5).

## 4. Discussion

The key findings of this study were that the informal WhatsApp teledermatology service is meeting a need and continues to grow (see Appendix A—Detailed Results and Table A1, Table A2, Table A3, Table A4 and Table A5). It benefits patients, referring doctors and dermatologists, and the users do not want to change to another app or system. Both doctors and dermatologists had limited awareness of current telemedicine guidelines in South Africa and ways of addressing LRE issues, particularly data security, consent, record-keeping, and storage. There was broad agreement amongst the two main stakeholder groups (KZ-N District Hospital doctors and KZ-N dermatologists) regarding the need for formalisation aligned with the pragmatic and collaborative development of relevant clinical and ethical guidelines, standard operating procedures, and technical guidance. Doctors and dermatologists were comfortable with an on-call dermatologist format for WhatsApp teleconsultations, which could be structured as a single point of contact with one dermatologist on-call for all, or a group of, district hospitals in KZ-N.

In the absence of any formal services and exploiting its ubiquity, low cost, and ease of use [31,32], doctors and specialists are using WhatsApp of their own volition. Spontaneous and informal use of WhatsApp by clinicians and community healthcare workers in clinical practice is common in both the developed world [24,32,33,34] and the developing world [18,31,35,36,37]. Despite such success, challenges exist, including a lack of Wi-Fi [38] and the issue of cost [18], with free Wi-Fi and apps being considered key to supporting the use of technology in healthcare [33]. In addition, authentication of both senders and recipients of transmitted healthcare information is of concern and is noted in the HPCSA guidelines [22] and published literature [26,39,40].

The application of WhatsApp (and other IM tools) for healthcare is rapidly growing globally. A simple PubMed search, “(WhatsApp OR “instant messag*”) AND (health OR healthcare)”, shows nearly 2000 citations, of which 16 are systematic reviews published in the past 5 years [24,41,42,43,44,45,46,47,48,49,50,51,52,53,54,55]. These systematic reviews confirm the global use, the breadth of application, and clinical specialities in which instant messaging (including WhatsApp) is used. However, no prior research has provided details of current practice in WhatsApp-based teledermatology nor offered evidence-based insight about how formalisation might be achieved. Such formality (e.g., policy, standard operating procedures, and practical guidelines) would ensure that the practice is conducted appropriately and be valuable for any current informal WhatsApp service anywhere in the world. The perceived benefits of WhatsApp use have been widely reported [34,38,56,57]. These include clinical efficiency through ready access to dermatologists [35,58,59], ease of use [33], favourable response times for on-call settings [19,57,60,61], reduced referral rates for face-to-face consultation [37,56,58], and passive learning [36,37,58].

In KZ-N, only 6% of patients referred via WhatsApp required a subsequent face-to-face consultation [19], and in India, a clinical diagnosis was made in all consults [35]. Satisfaction with WhatsApp use amongst doctors and dermatologists was high in this and other studies [19,56,62,63]. Other benefits noted were cost-effective consultation, improved patient outcomes [64], and reduced socio-economic costs of travel and lost work-time for patients [19,65].

As in the literature [36,37,58], doctors in this study reported the benefit of passive learning, some facilitated through access to expert advice and review of cases in chat groups (Appendix A, Theme 3—Utility—Benefits; Learning, education, and training; Table A3). Similarly, the dermatologists in KZ-N noted that doctors were becoming empowered to make their own diagnoses (Appendix A, Table A3). Fundamentally, however, many general practitioners around the world have limited dermatology training and knowledge. In the UK, “… dermatology training for GPs [General Practitioners] has been limited, with GPs lacking confidence in diagnosing and managing skin conditions.” [66], while in Saudi Arabia, 69.5% of primary healthcare physicians had insufficient knowledge of common skin disorders [67]. In Mali, healthcare workers made clear diagnoses and gave appropriate treatment in only ~42% of cases and prescribed unnecessary drugs in 52% of cases ([68]). 

Legal, regulatory, and ethical issues related to WhatsApp use, including record-keeping, data storage, privacy, and consent, remain of concern in many jurisdictions. A significant issue is the record-keeping of WhatsApp consultations, with few reports in the literature noting any concern or need for retention of original instant messaging text messages or photographs for audit purposes [69,70]. Some doctors in KZ-N transcribed the advice dermatologists gave them into the patient’s paper records, given the absence of electronic medical records (Appendix A, Theme 5—Poor Understanding of LRE Issues—*Record-keeping*). Others, including dermatologists, kept no formal paper or electronic record but retained some images and text stored on their phones as an informal, unauditable record (Appendix A, Theme 5—Poor Understanding of LRE Issues—*Record-keeping*), a situation common in other developing countries [61,71]. The doctors and dermatologists in this study felt they would recall the patient and consultation details later. However, a US study that assessed the ability of physicians to correctly identify their patients by reading de-identified discharge summaries (which included procedures, diagnoses, signs, and imaging results) during a period of about two months found that none of the 86 de-identified discharge summaries were correctly identified by any of the 19 physicians [72]. 

The literature offers clinicians no formal guidance on how to transfer and store WhatsApp communications [26]. Workarounds include printing the WhatsApp consult for filing in a patient record [73] or transferring WhatsApp consults to a computer for storage and record retrieval [70]. WhatsApp messages and attached images could be forwarded as encrypted emails to a centralised secure server [73] using the patient’s identification number as their identifier [22].

Proper patient identification throughout the continuum of care is essential to guarantee patient care and safety [74,75], and misidentification is a common source of harm [76]. The literature advises de-identifying or anonymising patient information shared electronically [22,69,77]. Most doctors in the study did not include patient identifiers or photographs of faces in their WhatsApp consultations (Appendix A, Table A5), but a traceable and auditable medical record can only be kept with the correct identification of a patient. The HPCSA provides conflicting guidelines. Section 5.8 of the Telehealth guideline speaks to “Required patient information to be included in electronic communications (e.g., name, identification number, and type of transaction)” [22], while Section 5.9.3d [22] and Section 6.5 of the HPCSA’s Ethical Guidelines on Social Mediarequire the removal of personal information in electronic communications [25].

Consent for sending a WhatsApp consultation and photographs is an unresolved issue [73,78,79]. Similar to the literature, consent in KZ-N was mostly verbal or implied, with written consent seldom obtained (Appendix A, Table A5). Some doctors recorded consent in the patient’s records. To confirm consent, it has been suggested that the referring doctor should send a photograph of the consent form together with the text message and photographs [80]. 

All key informants in the current study confirmed the need to formalise the process of using WhatsApp for dermatology consults (Appendix A, Theme 4—Process). This would include being officially sanctioned and given a definite structure, with necessary changes to referral pathways and procedures, and policy by KZ-N DoH. In addition, supporting guidelines for WhatsApp use would be needed, such as data security, record-keeping, storage, and patient consent (Appendix A, Table A5). 

Privacy is a primary concern when sharing patient information electronically, termed electronic protected health information, or ePHI [81]. Discussion of security and privacy issues related to ePHI is typically absent or only superficially acknowledged in the literature on smartphone use by clinicians [24]. Yet concerns are still raised, particularly in the developed world, that smartphone and IM use have the potential to compromise data privacy and data protection laws due to the exchange of identifiable healthcare data [24]. Given reasonable safeguards, this should not “diminish or disqualify WhatsApp as an acceptable tool in clinical and research setting[s]” [82], and WhatsApp was found to satisfy the needs of clinicians and patients in the developing world during the COVID-19 pandemic [83]. Furthermore, the preoccupation with the privacy of electronic data stems from rules originating with the Health Insurance Portability and Accountability Act of 1996 (HIPAA) [81], established almost 30 years ago—long before the common use of online and mobile services for healthcare and recent widespread legislation on privacy and data protection.

Similar to earlier studies in KZ-N [16,23], participants in this study were largely ignorant of the LRE issues related to telemedicine and the HPCSA guidelines (Appendix A, Table A5). Indeed, some doctors did not equate their use of WhatsApp with telemedicine [16]. Some concerns raised during the interviews included who assumed patient responsibility and the legality of teleconsultation, yet these are addressed in the HPCSA General Ethical Guidelines [22]. This lack of clinician awareness of laws and guidelines has been seen elsewhere [84,85], highlighting the need for awareness of issues [84,86], education [24,78], and training [3,87].

Although some doctors and dermatologists in the study expressed LRE concerns with WhatsApp, they still continued to use it (Appendix A, Table A2, Table A3, and Table A5). Similarly, global studies have shown that despite clinicians being aware of data privacy and protection laws, they may nonetheless choose to disregard them [34,88], often because of the benefits to the patient and physician [89]. Indeed, given current laws and regulations in the European Union and the USA, many IM apps (including WhatsApp) are considered “non-compliant” for telemedicine use in these jurisdictions [90]. Yet their use in these jurisdictions has been acknowledged [3,33]. This is in contrast to developing countries where, for example, WhatsApp has been designated as one of the most suitable tools for telemedicine [82,83]. Perhaps more important are pragmatic views. Stoklosa and colleagues note that “a blanket ban on the use of all app-based communication platforms risks their off-label use” and that this would place “patient’s privacy in far greater danger compared to finding ways to integrate this technology into clinical settings safely” [91]. Similarly, De Benedictis and colleagues state that “complete prohibition” of WhatsApp use would “be useless and counterproductive” [34]. 

Similar to other settings [31,38], participants in this study requested guidance on the LRE use of WhatsApp for inter-clinician dermatology (Appendix A, Table A5). Of particular concern in South Africa would be compliance with the Protection of Personal Information (POPI) Act [77] and HPCSA Guidelines [22] on the electronic exchange of healthcare information. Sharing of patient information is only considered ethical and legally justifiable when divulged to the medical team immediately responsible for treating the patient, provided the patient has consented to such sharing [92]. This would argue against using chat groups where most members would not be directly involved in a patient’s care. 

Globally, there are few guidelines for using instant messaging apps like WhatsApp [26,27,92]. Overarching guidelines like those of the HPCSA lack the granularity or practicality users require for clinical, technical, and operational guidelines. Consequently, busy clinicians have to assume an unclear data stewardship responsibility [93] and have uncertain defence in a social media context [92]. 

The insight gained from this study and the identification of particular issues that require urgent resolution will help the national and KZ-N Departments of Health understand what steps must be taken at the policy, process, and procedural levels to meet the need for a formalised WhatsApp teledermatology service in KZ-N. The findings have also led to the drafting of a policy brief to this end. 

Given the widespread and increasing use of IM apps such as WhatsApp by healthcare providers, these findings and the policy brief will have much broader value, being applicable to many clinical settings in other developed and developing nations.

## 5. Conclusions

This study has provided evidence-based insight into doctors’ and dermatologists’ perspectives and performance using WhatsApp for providing specialist dermatology consultation services. Such increasingly common practice should be embraced as forward-thinking and supported by governments as they seek alignment between policy-makers, regulators, and the needs of service providers to secure sustainable formalisation of these services. This would include the development of guidelines that address LRE requirements yet are practical and established collaboratively and with active and considered inputs from doctors, specialist clinicians, and patients.

## Figures and Tables

**Table 1:** Themes, sub-themes, and sub-sub-themes *(in italics)* identified from qualitative content analysis of KII transcripts.

Themes	Communication	Usability	Utility	Process	Poor Understanding of LRE * Issues
**Sub-Themes**	Initiation Influence Contacts Frequency Continuity Other specialities	Ease of Use Alternative IM apps Case history	Benefits Connectivity Status quo Learning Education Training Medication access	On-call dermatologist Formalising and scaling up What is wanted: *-* *Records* *-* *Accountability* *-* *On-call roster* *-* *Awareness* *-* *Guidelines*	Privacy Security Other LRE issues: *-* *Virtual consultation* *-* *Social media ‘abuse’* *-* *Chat groups* *-* *Responsibility for patient care* *-* *Anonymity/de-identification* *-* *Consent* *-* *Record-keeping* *-* *Guidelines—HPSCA*

* Legal, regulatory, and ethical.

## Data Availability

Dataset available on request from the authors.

## Abbreviations

DoH	Department of Health
ePHI	Electronic Protected Health Information
HPCSA	Health Professions Council of South Africa
KZ-N	KwaZulu-Natal
LRE	Legal, Regulatory, Ethical
POPI	Protection of Personal Information Act

## Appendix A. Results

### Appendix A.1. Theme 1—Communication

This theme concerned comments about the activity and people involved in communicating with one another to give or receive specific information. Subthemes included ‘initiation’ of WhatsApp use, ‘influence’ regarding initial introduction to use, ‘contacts’ made and by whom, the ‘frequency’ and ‘continuity’ of communication, and WhatsApp use for ‘other specialities’.

Initiation. Dermatologists initiated most of the links with district hospitals with most doctors, and the remaining dermatologists were introduced to WhatsApp use by their colleagues, particularly after difficulties were experienced with a prior videoconferencing service (Appendix A, Table A1, Dr 8, 9, Derma 2, 3, 4). Initially, some dermatologists had requested doctors to send photographs and a consultation history until the practice was commonplace. Some indicated they need not ask as this is often done routinely; others indicated they might ask for a history following receipt of images or vice versa.

Influence. All 12 doctors had recommended using WhatsApp for teledermatology to others, with half having recommended it to more than 10 colleagues (Appendix A, Table A1, Dr 9).

Contacts. All dermatologists received referrals from doctors at district hospitals, with some receiving messages from up to 18 hospitals. No specific telemedicine outreach programme was in place linking dermatologists to specific hospitals. On occasion, they did not know who was sending the case. Some doctors noted difficulties in knowing who to call and therefore needed to identify the dermatologist on-call via the dermatology clinic at the KZ-N Medical School or the hospital switchboard (Appendix A, Table A1, Dr 3), while others had a dermatologist to whom they regularly referred.

Frequency. The number of referrals had increased over time, and this was attributed, in part, to the cancellation of the provincial flying doctor outreach programme in which the dermatologists had participated and, most recently, the COVID-19 pandemic. The current volume of cases varied from fewer than 10 cases per week (*n* = 8) to two dermatologists receiving between 41 and 50 referrals per week. The number of cases was highly variable; for example, receiving five in one day when five in a week was the norm.

Continuity. All respondents confirmed their wish to continue using WhatsApp, and most noted that the informal WhatsApp service had led to the development of a relationship between the doctors and the dermatologists.

Other specialities. All 12 doctors confirmed that WhatsApp was used for several other specialities, including obstetrics, gynaecology, ophthalmology, orthopaedics, radiology, internal medicine, paediatrics, wound care, burns, sharing results of investigations such as blood tests, and administrative purposes.

**Table A1:** Selected quotes from doctors and dermatologists for Theme 1 (Communication) concerning the sub-themes ‘Initiation’, ‘Influence’, and ‘Contacts’.

**Doctors**
Dr 3 (Contacts): *“Yes, it takes time to call and find out who is on call.”* Dr 8 (Initiation): *“Yes, so they said* [use] *the dermatology* [chat] *group that we have with the doctors in the hospital and specialists from Durban.”* Dr 9 (Initiation): “*Yes, so I was initially introduced to this through colleagues.”* Dr 9 (Influence): *“I actually introduced all the new doctors, who would start there, to the* [chat] *group as well.”*
**Dermatologists**
Derma 2 (Initiation): *“Yes, we just started using WhatsApp and asked the doctors to send us pictures.”* Derma 3 (Initiation): *“Before I joined the department, there was a mechanism in place …. full-on teledermatology* [videoconferencing]. *But depending on signal …. we often get cut off. What we started to do was when doctors or nurses would call through from hospitals and give us the history, then we’d ask* [them] *to send photographs of the patient, as we wanted to make an assessment.”* Derma 4 (Initiation)*: “I first began by using the telderm service* [videoconferencing] *and was later introduced by my colleagues to the WhatsApp group in 2019.”*

### Appendix A.2. Theme 2—Usability

Comments regarding usability referred to the ease of use of WhatsApp to present a case history and clinical photographs. Sub-themes included: ‘Ease of use’, ‘Alternative IM apps’ used, and ‘Case history’.

Ease of use. All doctors found WhatsApp easy to use for teledermatology and noted benefits such as the immediacy of use and response (n = 8), access to expert advice (6), familiarity with its use (6), its ubiquity (2), ability to upload images/video (2) and the ability to track messages (monitoring progress) (1). Any difficulties identified by doctors related not to the app but to external factors: connectivity (9), inconsistent electrical supply (1), poor lighting affecting image quality (1), and picture upload time (1). Three doctors did not identify any problems. Only two doctors mentioned difficulties with attaching and sending images, one in editing the picture for de-identification and another reporting phone storage capacity issues. Typing difficulties and autocorrect text challenges were reasons for two doctors’ preference for sending a voice note (Appendix A, Table A2, Dr 7).

Alternative IM apps were used. All doctors and dermatologists confirmed their intention to stay with WhatsApp rather than change to another instant message app (Appendix A, Table A2, Dr 10, Derma 4, 9). One dermatologist mentioned using Vula (an alternative telemedicine application in South Africa) but also commented on difficulties with its use (Appendix A, Table A2, Derma 14). One dermatologist continued to use WhatsApp but desired a better application without specifying in what way.

Case history. Most doctors (n = 10) had no difficulty in presenting the case history or knowing how much detail to include and were comfortable with what was sent (Appendix A, Table A2, Dr 4), although it was noted that on occasion, the dermatologist requested extra information (Appendix A, Table A2, Dr 8). One doctor first discussed the case with the dermatologist telephonically before sending the WhatsApp message and photographs, and another expressed concern about the adequacy of the history and whether this constituted a breach of confidentiality.

**Table A2:** Selected quotes from doctors and dermatologists for Theme 2 (Usability) concerning the sub-themes ‘Ease of use’, ‘Alternative IM apps’, and ‘Case history’.

**Doctors**
Dr 4 (Case history): *“No, it was not difficult to not have too much information to include* [in the case history]*.”* Dr 7 (Ease of use): “*The best way to do it is just to send a voice note where you can present the patient.”* Dr 8 (Case history): *“I think we’re all pretty used to doing it* [case history] *through text nowadays*, *and also if there’s things missing, the platform is dynamic, so the consultant asked questions and we gave further answers.”* Dr 10 (Alternative IM apps): *“No, I’m still using it* [WhatsApp]*. I would not consider using any other app.”*
**Dermatologists**
Derma 4 (Alternative IM apps used): *“Yes, we’re happy to continue with WhatsApp …”* Derma 9 (Alternative IM apps used): *“I think, to be honest, for now, WhatsApp works really well, nothing complicated …”* Derma 14 (Alternative IM apps used): *“But like I said when I left the previous department, we used an app* [Vula] *but it was taking too much work. It wasn’t* [an] *easy thing or a way to do it, it felt like there’s just too much downloading.”*

### Appendix A.3. Theme 3—Utility

Utility refers to comments noting the usefulness of WhatsApp (e.g., benefits clinical practice and patient care, cost-effective). Sub-themes included ‘Benefits’, ‘Connectivity’, ‘Status-quo’, ‘Learning, Education, Training’, and ‘Medication Access’.

Benefits. All doctors identified benefits to themselves and their patients: clinical efficiency (n = 12), avoiding unnecessary patient travel (9), access to expert advice (6), efficient patient management (quicker diagnosis or referral) (6), avoiding unnecessary patient costs (2), diagnostic accuracy with pictures enhancing the referral and providing more information than just a telephone call (2), and passive education through the interaction with the dermatologist (2) (Appendix A, Table A3, Dr 10).

Connectivity. Only one doctor had Wi-Fi access for WhatsApp use provided at their hospital. The remainder used data on their personal mobile phone package and accepted the costs they incurred as it was for the ‘greater good’. It was noted that the landlines had been down at one hospital for “almost two years” (Appendix A, Table A3, Dr 9). The doctors did not know how much it cost to send their WhatsApp referrals and photographs.

Status quo. Doctors and dermatologists reported their strong universal desire to continue using WhatsApp (Appendix A, Table A3, Dr 2, 3, 5, Derma 11) but noted legal, regulatory, and ethical concerns with its informal use.

Learning, Education, and Training. Doctors acknowledged their poor knowledge of dermatology (Appendix A, Table A3, Dr 2, 11a), which was confirmed by the dermatologists (Appendix A, Table A3, Derma 8). Several doctors mentioned passive learning through their WhatsApp interactions with dermatologists (Appendix A, Table A3, Dr 11b) and the need for dermatologists to be non-judgemental. They also learnt through following the cases dealt with in chat groups (Appendix A, Table A3, Dr 8). Dermatologists also noted the educational benefit of the referrals to the doctors (Appendix A, Table A3, Derma 2).

Medication access. The dermatologists had mixed experiences with the availability of medications they prescribed for patients at district hospitals. Five said they knew what medications were available, with eight noting that medications were not always available. In the absence of onsite medication, alternative medications were prescribed, or prescriptions were provided for out-of-pocket purchases at private pharmacies, and four dermatologists reported having to resort to referral to the academic hospital to obtain medication.

**Table A3:** Selected quotes from doctors and dermatologists for Theme 3 (Utility) concerning sub-themes ‘Benefits’, ‘Status Quo’, and ‘Learning, Education and Training’.

**Doctors**
Dr 2 (Status quo): *“Service has been extremely consistent over many years, so I’ve been very impressed with it, you know. So, I’ll be very reluctant to change it to something that may not work ...”* Dr 2 (Learning, education and training): “*You know, it’s just that I don’t know myself enough about dermatology.”* Dr 3 (Status quo): *“… you are not going to find any willing buyer / willing seller agreement here, where any of us take down our WhatsApps. I can’t imagine that happening, ever.”* Dr 5 (Status quo): “*I don’t want this feature* [WhatsApp] *to be taken away.” * Dr 8 (Learning, education and training): “*… you can also learn* [from chat groups] *when other colleagues send pictures; you kind of learn from them even though you are not directly involved.”* Dr 9 (Connectivity): “*I’m so used to it by now because our phone lines, our landlines have been down for like almost two years now. So I’ve been using my own data in my own time discussing new patients. Look it’s what you have to do, it needs to be done.”* Dr 9 (Learning, education and training): *“… dermatology is always something that you always ask a second opinion on.”* Dr 10 (Benefits): *“… it was good to get things across and to get initial treatment started and give a plan to the patient.”* Dr 11a (Learning, education and training): “*Dermatology is not something we know a lot about.”* Dr 11b (Learning, education and training): *“My knowledge is growing getting an expert response* within a *short space of time.”*
**Dermatologists**
Derma 2 (Learning, education and training):*“… because we empower a doctor on the other side, we educate the doctors.”* Derma 8 (Learning, education and training): “*My colleagues were medical officers in the rural areas. They will try and describe something,* [but] *of course not everyone understands dermatology very much; they will try, and say ‘rash’ to me. Rash doesn’t say anything, so WhatsApp me. Then they send you the pictures and the consults.”* Derma 11 (Status quo): *“… no I think because everyone already has WhatsApp, I think if you guys are going to create an app or create something, it’s going to be problematic.”*

### Appendix A.4. Theme 4—Process

Process refers to the actions or steps taken to achieve a particular end. For WhatsApp teledermatology consultation, this begins with contacting a dermatologist, ideally through a formalised and scaled procedure. Sub-themes included ‘On-call dermatologist’, ‘Formalising and scaling up’, and ‘What is wanted’.

On-call dermatologist. According to the established referral pathway, doctors were required to contact the dermatologist on-call via a landline call prior to referring a patient to the dermatology clinic (Appendix A, Table A4, Dr 12). Because landlines were often out of order, doctors would sometimes simply initiate a cell phone discussion, and the dermatologists would request the doctors to send the patient’s history and relevant photographs using WhatsApp.

Most doctors (n = 10) and dermatologists (10) felt that having an on-call dermatologist for WhatsApp referrals would be useful, citing various reasons (Appendix A, Table A4, Dr 4, 10, Derma 1). All but one doctor (Appendix A, Table A4, Dr 5) would be willing to send consults to a Department of Health dermatologist they did not know, and most dermatologists (11) would be happy to receive consults from doctors they did not know, with discomfort expressed by the remainder (Appendix A, Table A4, Derma 11,13).

Formalising and scaling up. All doctors and dermatologists confirmed the need and desire for formalising the process of using WhatsApp for dermatology (i.e., officially sanctioned and given a definite structure), although some concern was expressed about conflict with the current referral process (Appendix A, Table A4, Dr 12). In terms of scaling, it was also considered important to get the DoH to formalise the service and expand it to all district hospitals (Appendix A, Table A4, Dr 1, 2, 12).

What is wanted. Doctors identified the need for a centralised service with a single number, a WhatsApp group for every hospital, a method for uploading written consent, and possibly extending the service to rural clinics. Some dermatologists’ suggestions also included rules for the deletion of photos, an email account on the hospital server, and a secure platform with chat group functionality. Dermatologists stressed regulatory/medico-legal compliance and the need for guidelines and standard operating procedures for WhatsApp teledermatology (Appendix A, Table A4, Derma 3, 4). These should address record-keeping (Appendix A, Table A4, Derma 13), data security and privacy, data/image storage, consent, responsibility for patient care, and referral pathways for those requiring in-person consultation. Both groups expressed the need for improved connectivity, free Wi-Fi access, and raising awareness of the availability of such a service.

**Table A4:** Selected quotes from doctors and dermatologists for Theme 4 (Process) concerning sub-themes: ‘On-call dermatologist’, ‘Formalising and scaling up’, and ‘What is wanted’.

**Doctors**
Dr 1 (Scaling up): *“Yeah, certainly the district hospitals for sure.”* Dr 2 (Scaling up): “*I think it will be very useful for all district hospitals to have access to a dermatology referral for gaining advice.”* Dr 4 (On-call dermatologist)*: “I should say having a centralised service with a number, yeah someone accessible …”* Dr 5 (On-call dermatologist)*: “I would not* [… be willing to send consults to an on-call dermatologist I do not know].” Dr 10 (On-call dermatologist): *“I think it’s just a more formal way if someone is on-call and, you know, you can WhatsApp so you don’t have to call ahead first and we can just WhatsApp and someone will WhatsApp back fairly quickly. That would be helpful.”* Dr 12 (On-call dermatologist/Formalising and scaling up): *“Remember, a doctor cannot just call any hospital without it being part of the referral pathway. So now we’re calling and consulting at some form of university, it’s not part of our referral pathway….”* Dr 12 (Formalising and scaling up): *“I think that it needs to be approved by a higher authority, you know. I’m always thinking of litigation things like that. So if it is going to be formalised, the Department of Health will really need to come to the party and give approval for this to happen of course.”*
**Dermatologists**
Derma 1 (On-call dermatologist): *“So that we can respond to all consults timeously, arrange leave.”* Derma 3 (What is wanted): “*I think proper guidelines and* [a] *system with the proper steps to follow in terms of what information we need to provide, maintaining patient confidentiality and legal requirements, how information needs to be stored, how long it needs to be stored, and providing a safe platform that we are able to communicate* [with]*.”* Derma 4 (What is wanted): *“It’s very important that we have guidelines.” * Derma 11 (On-call dermatologist): “*We try not to do that so much* [respond to someone we do not know] *unless it’s really an emergency*.” Derma 13 (On-call dermatologist): *“From outside other centres who are not in my drainage* [referral] area, no, I would prefer not, no.” Derma 13 (What is wanted): *“We need something that is more formalised so we can keep patient records …. I mean if I delete my WhatsApp, then it means all that information is lost for some people. We don’t even* [have] *back up* [of] *our consults.”*

### Appendix A.5. Theme 5—Poor Understanding of LRE Issues

Legal and regulatory issues concern laws and rules that govern or impact the conduct of individuals within healthcare systems, whilst ethical issues involve rules or standards that (imposed through professional entities or social norms) govern the conduct of members of a profession. Sub-themes were ‘Privacy’, ‘Security’, and ‘Other LRE issues’. Notable sub-sub-themes were ‘Responsibility for patient care’, ‘Consent’, ‘Record-Keeping’, and ‘Guidelines—HPSCA’.

Privacy. Issues were raised related to storing data (intentional saving for purposes of maintaining a record) versus unnecessary or inadvertent retention of sensitive data on phones when no longer required and compliance with the South African Protection of Personal Information (POPI) Act. Most doctors and dermatologists stored patient information and photographs on their phones for later reference. Concerns were raised about the legality of retaining these data or the breach of patient privacy if the phone is lost. Possible breaches of confidentiality were alluded to, with concern about inadvertently sending messages to the wrong person.

Security. Security issues concerned comments about mechanisms by which data are protected against unauthorised access and loss or corruption at any point in the ‘data lifecycle’. Nine doctors were aware of the security features of WhatsApp, including end-to-end encryption, and platform security, and some had concerns that Facebook had access to their contacts. Most doctors (9) and six of the dermatologists said they were aware of the security and privacy features of WhatsApp, but their explanations revealed misconceptions and limited understanding of the WhatsApp data transmission process and security features.

Other LRE issues. Most doctors (9) and dermatologists (11) indicated they had LRE concerns about using WhatsApp (noting liability and breach of privacy), but they continue to use it. All doctors and dermatologists felt there should be guidelines for using WhatsApp (Appendix A, Table A5, Dr 7, 8, Derma 4, 9). Comments were also made about (i) the legality of virtual consultation, (ii) social media abuse, (iii) chat groups, (iv) responsibility for patient care (Appendix A, Table A5, Derma 1), (v) anonymity and de-identification (Appendix A, Table A5, Dr 3, 6, 12), and (vi) authentication. Many of the responses again highlighted a poor understanding of the HPCSA guidelines. Responses to questions concerning LRE matters indicated misunderstandings regarding many issues.

*Consent.* Only one doctor gained written consent for sending WhatsApp consults with photographs (Appendix A, Table A5. Dr 3), with ten reporting that consent was verbal (Appendix A, Table A5. Dr 10), and one that it was “implied verbal”. Only half (n = 6) kept a record of consent that was obtained verbally. Nine dermatologists had no idea if consent was obtained (Appendix A, Table A5, Derma 2, 3), three others felt it was not obtained, and two others thought that consent was obtained.

*Record-keeping.* Given the absence of electronic medical records in the district hospitals, record-keeping was paper-based. Written records were reportedly kept by five doctors and one dermatologist. In an apparent contradiction, most doctors (n = 10) and dermatologists (10) stored ‘consults’ (often only the images) on their phones (Appendix A, Table A5, Dr 3, 7, 11, Derma 3, 4, 6). One doctor did report transcribing the management plan received from the consultant into the patient’s notes before deleting the message from their phone (Appendix A, Table A5, Dr 6). Doctors said that the information recorded did not include patient identifiers (Appendix A, Table A5, Dr 11, 12), but sometimes did disclose age, gender, and patient images. Whether written or electronic, record-keeping concerns relate to the privacy and security of these records. No doctor or dermatologist noted any issue regarding patient confidentiality, such as gaining consent to share information in a chat group.

*Guidelines—HPSCA.* Nine doctors were aware of the HPCSA General Ethical Guidelines for Good Practice in Telemedicine. Many responses indicated that they had not read the Guidelines, and no doctors said that they were following any guidelines. Most dermatologists were unfamiliar with the HPCSA Guidelines. Only one had looked at the guidelines, and another knew they existed. Concern was expressed about any guidelines being effective in real-life circumstances (Appendix A, Table A5, Derma 14).

**Table A5:** Selected quotes from doctors and dermatologists for Theme 5 (Poor Understanding of LRE Issues) concerning sub-sub-themes*: ‘Responsibility for patient care’, ‘Anonymity and de-identification’, ‘Consent’, ‘Record-keeping’, and ‘Guidelines—HPCSA’*.

**Doctors**
Dr 3 (Record-keeping): “*I worry if we keep photographs on the phone and the other doctors keep photographs. He’s using a phone for another reason, not medical at all, and there are multiple pictures of patients.”* Dr 3 (Anonymity and de-identification): “Yes, w*e try as far as possible to not have identified features …”* Dr 3 (Consent): *“The fact is that we do use WhatsApp for dermatology, and we get the patient’s consent. We get signed consent and insert into the files, and then we check that we have the right to send it.”* Dr 6 (Anonymity and de-identification/Record-keeping): *“Maybe sometimes the age might be important, gender, but other than that, patient identifiers are not usually important in the medical history. But in the patient’s file, all this information is there, so I basically record the advice of the specialist, and I put* [it] *in the patient’s file and I delete the WhatsApp.”* Dr 6 (Record-keeping): *“No, because remember, after the consult usually I’ll transcribe whatever advice you get into the patient’s file, and I usually delete.”* Dr 7 (Guidelines—HPCSA): *“In* [the] *long run it is* [need for guidelines] *because you don’t want to open up yourself to medico-legal suits or questions about* [whether] *what you are doing is safe and ethical and in the best interests of the patient. I think we use the tech in everybody’s best interest and we need to find a safe way that we can use it that is ethical and protects the patient and consultant doctor..”* Dr 7 (Record-keeping): *“I think everybody* [keeps a record] *on the phone; keeps it, stays on the phone.”* Dr 8 (Guidelines—HPCSA): *“Yeah, I think it’s necessary to protect for the safety of the patient and to have guidelines and to know how to use them, as long as they are appropriate, …”* Dr 10 (Consent): *“I ask for consent to take a picture, yeah, and I tell them what I’m going to do with it, and I told* [them] *that I hide their faces unless obviously it’s something on the face. Yes, I do tell them that for what reason it is, and I get their consent.”* Dr 11 (Record-keeping/Anonymity and de-identification): *“… I don’t know how legal it is to keep pictures of patients on the phone. I mean the phone could get lost or stolen and someone could extract the images off the phone. I try to make a point that there are no faces or identity.”* Dr 12 (Anonymity and de-identification): *“But there is a picture and you don’t know who it is. Doctors do not write a name.”*
**Dermatologists**
Derma 1 (Responsibility for patient care): “*I have concerns that if there were litigation, I am not protected. If they sent you a text, the ball is now in your court, right, and now you become responsible for the consults, which for me is something that shouldn’t be, …”* Derma 2 (Consent): “*I hope they are gaining consent on their side, so at least sending me pictures is not an illegal thing.”* Derma 3 (Record-keeping): *“… it’s not a formal record but I sort of keep it on my phone in terms of need to know … I don’t do it for all the patients.” * Derma 3 (Consent): *I have no idea. I think in the past, we had not thought about it much, but with the POPI* [Act] *we are quite careful with it now. We have to ask them for their consent.”* Derma 4 (Guidelines—HPCSA): *“It’s very important that we have guidelines.”* Derma 4 (Record-keeping): *“So I keep records by storing the images on my phone.”* Derma 6 (Record-keeping): *“You know for myself I don’t keep records. Some management plans are on my phone, but for me, it’s easier when once they’ve actually sent the pictures in the consult through WhatsApp I just take my phone and just call them.”* Derma 9 (Guidelines—HPCSA): *“I think that’s one thing that we as doctors sometimes tend to overlook to help patients, but they* [guidelines] *are definitely needed.”* Derma 14 (Guidelines—HPCSA): “*Definitely something that is practical, but I found some of these guidelines* [are] *drafted by someone sitting in an office. In practice, it’s not easy for us to actually use them. So, they have to be very, like, to the point and practical. I wish that the process could be consultative, so sort of guidelines, but it has to be practical for all stakeholders including patients.”*
